# Obesity parameters in relation to lung function levels in a large Chinese rural adult population

**DOI:** 10.4178/epih.e2021047

**Published:** 2021-08-03

**Authors:** Xiang Zeng, Dongling Liu, Zhen An, Huijun Li, Jie Song, Weidong Wu

**Affiliations:** 1 School of Public Health, Xinxiang Medical University, Xinxiang, China; 2 Laboratory of Environmental Medicine and Developmental Toxicology, Guangdong Key Laboratory of Environmental Pollution and Health, School of Environment, Jinan University, Guangzhou, China; 3 Henan Key Laboratory of Medical Tissue Regeneration, Xinxiang Medical University, Xinxiang, China

**Keywords:** Obesity, Body fat distribution, Overweight, Pulmonary function, Rural area, Chinese

## Abstract

**OBJECTIVES:**

The association between obesity parameters and lung function indicators in the general Chinese rural adult population remains unclear.

**METHODS:**

In total, 8,284 Chinese adults aged 20 years to 80 years old from Xinxiang were recruited. Obesity-related parameters, including body mass index (BMI), waist circumference (WC), hip circumference (HC), waist-to-hip ratio (WHR), waistto-height ratio (WHtR), body fat percentage (BFP), basal metabolism, and visceral fat index, and lung function parameters such as forced vital capacity and forced expiratory volume in first second were measured.

**RESULTS:**

The total prevalence of obesity defined by BMI, WC, WHR, WHtR, and BFP was 23.2%, 58.2%, 66.7%, 69.2%, and 56.5%, respectively. Spearman correlation analyses showed significant correlations between all obesity-related parameters and lung function. Linear regression analyses further demonstrated that BMI, WHtR, BFP, and general obesity defined using those indicators were negatively associated with lung function, while WC, WHR, and central obesity defined accordingly were positively associated with lung function. The relationship between general obesity and lung function was more evident in women than in men, while the link between central obesity and lung function was more obvious in men than in women.

**CONCLUSIONS:**

Obesity is closely related to lung function in the general Chinese adult population. Weight control and loss are important strategies to improve lung function and respiratory health.

## INTRODUCTION

Obesity is a significant global public health challenge; its prevalence has grown considerably in recent years, to the point that it has become a global pandemic accompanied by tremendous morbidity and mortality. It is estimated that more than one-third of adults are overweight or obese around the world [1], and the global prevalence of obesity will be 1.12 billion in 2030 [2]. Obesity and overweight caused approximately 3.4 million deaths worldwide in 2010 [3]. The epidemic of overweight and obesity is a major public health problem in China, although the problem is less severe than in developed countries [4-6]. In urban regions of China, the overall prevalence of overweight ranges from 15.7% to 41.8%, and the prevalence of obesity ranges from 6.3% to 19.5% in adults [7-9]. Obesity is an important risk factor and modifier for metabolic disorders, cardiovascular disease, dyslipidemia, asthma, and cancer [10-12]. Previous studies have demonstrated that obesity may cause restrictive ventilation dysfunction by reducing lung and chest wall compliance due to excessive fat deposits in the diaphragm, chest wall, and abdominal cavity [11-15]. In addition, weight loss and exercise can help patients with obesityrelated lung disease reduce the resistance to respiration caused by excess fat [16-18]. However, relatively little is known regarding the detrimental influence of obesity on non-asthmatic respiratory conditions, particularly respiratory lung function and damage in the general Chinese rural population.

There are several commonly used indicators that reflect body fat distribution and define obesity, including body mass index (BMI), waist circumference (WC), hip circumference (HC), waist-to-hip ratio (WHR), waist-to-height ratio (WHtR), body fat percentage (BFP), basal metabolism (BM), and visceral fat index (VFI) [19-23]. BMI has been frequently used to define general obesity, while WC, WHR, WHtR, and BFP have been commonly used to define abdominal obesity. Briefly, general obesity is defined as a BMI ≥ 25 kg/m^2^ according to the World Health Organization recommendations for Asians [24]. In contrast, abdominal obesity (clinically known as central obesity) is defined as WC ≥ 90 cm for men and WC ≥ 80 cm for women, WHR ≥ 0.90 for men and WHR ≥ 0.80 for women, WHtR ≥ 0.5 both for men and women, or BFP ≥ 25% for men and BFP ≥ 33% for women [19,23,25-27]. A strength of general obesity as a measure is that it is useful for classifying the severity of obesity, but it has weaknesses in terms of specificity, as it cannot distinguish between fat mass and lean mass or denote the pattern of regional fat distribution. Instead, abdominal or central obesity is closely related to central fat localization and all-cause mortality, independently of general obesity [28]. The deposition of fat in the thorax, abdomen, visceral organs, and an apple-like body shape are the main characteristics of central obesity. Systemic or peripheral obesity is characterized by the deposition of fat in the hips, thighs, limbs, subcutaneous tissue, and a pear-like body shape. The distinction between different types of obesity is important since central obesity tends to have a more direct impact on lung mechanics and metabolic inflammation than peripheral obesity [11]. Several feasible mechanisms have been proposed to explain how obesity affects respiratory impairment, such as decreased total respiratory system compliance, increased airway resistance, reduced lung volume, and altered ventilation and gas exchange [4,11,13].

Inconsistent relationships between obesity and lung function have been reported in studies of populations with different conditions and backgrounds [29-39]. For instance, there is a higher metabolic risk in Asians than in Caucasians at a given BMI level [37]. To date, there is still a lack of comparative studies analyzing the relationship between different obesity parameters and lung function within the same study. Therefore, we conducted a cross-sectional study on rural residents of China, analyzed big data, and aimed to further confirm the relationship between several obesity parameters and lung function indicators in the adult population in central China. Clinicians should be alert to the possible adverse effects of obesity on lung function, and weight control and body shape management should be addressed in the prevention and improvement of respiratory disease.

## MATERIALS AND METHODS

### Study sites and participants

This study was conducted in rural areas in Xinxiang County, Henan Province, China (Supplementary Material 1). The inclusion criteria were subjects with no acute disease or hospitalization experience within 1 month who were not pregnant during the period of investigation. A total of 8,375 subjects aged 20-80 years met the inclusion criteria and were subsequently screened from several villages in 2 towns (Qiliying and Langgongmiao) in Xinxiang County using a cluster sampling method (Supplementary Material 1). Ninety-one subjects were excluded because that they were unable to complete the lung function test. Therefore, 8,284 adults were finally included in the analyses of this study. The participants completed a general health questionnaire and underwent a routine physical examination.

### Questionnaire

Participants were required to complete a questionnaire through face-to-face conversations with trained staff. Physical measurements were conducted using standardized procedures as described below. The questionnaire collected basic individual information including age, gender, marital status, residential address, income, education, and lifestyle characteristics (e.g., smoking status and alcohol consumption). The physical examination parameters included body height, weight, WC, HC, BFP, BM, and VFI [19].

### Physical measurements

All participants underwent weight, height, waist, hip, BFP, BM, and VFI measurements according to a standard protocol [20]. The physical examination was performed after participants had taken off their shoes and heavy clothes. Weight was measured to the nearest 0.1 kg, and height was measured to 1 decimal point. WC was determined to the nearest 0.5 cm around the abdomen at the level of the umbilicus/belly button and HC. HC was read to the nearest 0.5 cm at the fullest part between the abdomen and groin. BMI was defined as weight (kg) divided BM, by height squared (m^2^). WHR was calculated as WC divided by HC. WHtR was calculated as WC divided by height. BFP, and VFI were measured using Omron HBF-371 (Omron, Kyoto, Japan) body fat and weight measurement scales using bioelectrical impedance analysis. Specifically, human body resistance was measured by a weak current flowing through hands and feet, and the characteristic of the human body. In other words, human issue with more water is easy to conduct electrcity, while adipose tissue is hardly conductive. BM was automatically calculated using the formula provided with the scales, which mainly depend on gender, weight and body fat. BFP was defined as the body fat weight divided by total weight. All measurements were taken twice and the average of the 2 values was used in further analyses [19].

### Definition of obesity

General obesity was defined as a BMI ≥ 25 kg/m^2^ [24,33]. Central obesity was defined as WC ≥ 90 cm for men and WC ≥ 80 cm for women, WHR ≥ 0.90 for men and WHR ≥ 0.80 for women, or WHtR ≥ 0.5 for both men and women [23]. Obesity was defined as BFP ≥ 25% for men and BFP ≥ 33% for women [27]. VFI was divided into four categories (from “thin” to “high”) according to the criteria used in a previous study, respectively [40].

### Spirometry

The lung function test was conducted with a portable spirometer (Chestgraph HI-801; Chest MI, Tokyo, Japan) with participants in a standing position following the standardized procedures of the American Thoracic Society criteria, with at least 3 measurements. The analyses used the highest value of forced vital capacity (FVC), forced expiratory volume in the first second (FEV_1_), vital capacity (VC), inspiratory capacity (IC), residual volume (RV), tidal volume (TV), expiratory reserve volume (ERV), inspiratory reserve volume (IRV), total lung capacity (TLC), peak inspiratory flow (PIF), peak expiratory flow (PEF), and peak expiratory flow time (PEFT) [41]. The spirometer was calibrated before each test according to the manufacturer’s instructions. The predicted values for FVC and FEV_1_ were calculated based on the equations for the Chinese adult population [42]. The main outcome variables were lung function and its predicted values, as well as restrictive respiratory defects [43].

### Statistical analysis

All data were entered using EpiData version 3.0. The statistical analysis was performed using IBM SPSS version 22.0 (IBM Corp., Armonk, NY, USA). Normally distributed continuous variables were presented as the mean± standard deviation and compared using the t-test. Non-normally distributed quantitative data were displayed as median and interquartile range and compared using the Mann-Whitney U test. Categorical variables were expressed as percentages and were analyzed using the chi-square test or Fisher exact test as appropriate. Spearman correlation analysis was used to explore the correlations between obesity and lung function parameters. Multivariate linear regression analyses were further carried out to evaluate the associations between obesity and lung function. Potential confounders were included in each full model when the p-value of the confounder was lower than 0.05. A p-value < 0.05 was considered to indicate statistical significance.

### Ethics statement

Participants provided written informed consent. The study was approved by the Medical Ethics Committee of the Xinxiang Medical University, Xinxiang, China. This study was conducted ethically in accordance with the World Medical Association Declaration of Helsinki. All participants provided written informed consent before enrollment and data collection.

## RESULTS

### General characteristics of the study population

The average age of the participants was 52.12± 12.42 years for men and 51.23± 12.09 years for women. The proportion of men and women was 40.2% and 59.8%, respectively. As shown in Table 1, men had higher current smoking and drinking rates, family income, and educational levels than women (Table 1). There was no significant difference in BMI between men and women (Supplementary Material 2). However, the height, weight, WC, HC, WHR, BM and VFI of men were higher than those of women, whereas the WHtR and BFP of men were lower than those of women (Table 2).

### Prevalence of obesity

**Figure 1.**
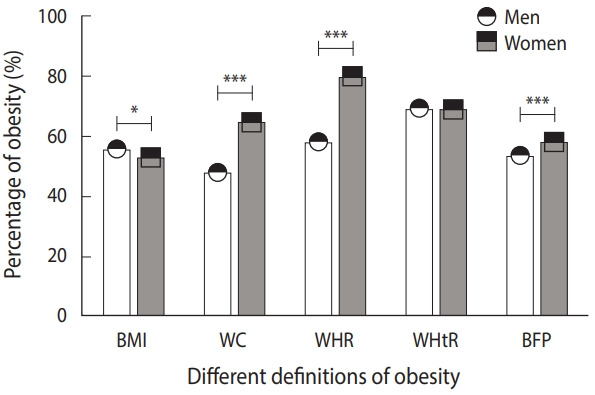
Comparison of the prevalence of obesity between men (n=3,327) and women (n=4,957). BMI, body mass index; WC, waist circumference; HC, hip circumference; WHR, waist-to-hip ratio; WHtR, waist-to-height ratio; BFP, body fat percentage. The average percentages of obesity are displayed as mean±standard deviation, and the error bar in the figure represents the standard deviation. ^*^p<0.05, ^***^p<0.001.

The total prevalence of obesity as defined by BMI, WC, WHR, WHtR, and BFP was 23.2%, 58.2%, 66.7%, 69.2%, and 56.5%, respectively. In addition to the prevalence of obesity defined by BMI and WHtR, which are height-related parameters, the prevalence of obesity defined using several other indicators such as WC (65.0%), WHR (80.2%), and BFP (58.3%) was higher in women than in men (48.0, 58.4, and 53.7%), respectively (Figure 1). In total, 1,891 (22.8%) participants had both general and central obesity. In other words, the prevalence of obesity defined by BMI and WHtR was not higher in women than in men, whereas the prevalence of obesity defined by WC, WHR, and BFP was higher in women than in men (Figure 1) (Supplementary Material 3). WC, WHR (as a parameter positively related to WC), and obesity defined accordingly were higher in women than in men. Instead, BMI and WHtR (as parameters negatively related to height) and obesity defined accordingly were higher in men than in women. Therefore, women’s higher WC and shorter height may contribute to their high prevalence of obesity when compared with men.

### Lung function levels

**Figure 2.**
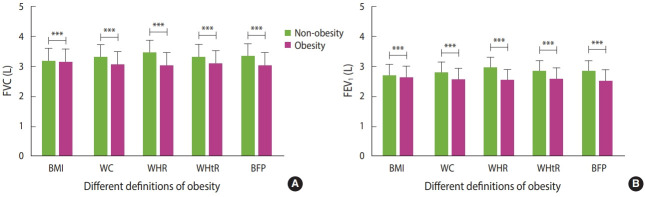
Comparison of FVC (A) and FEV_1_ (B) between non-obese and obese subjects. FVC, forced vital capacity; FEV_1_, forced expiratory volume in 1 second; BMI, body mass index; WC, waist circumference; WHR, waist-to-hip ratio; WHtR, waist-to-height ratio; BFP, body fat percentage. The comparison of FVC and FEV_1_ between obesity and non-obesity groups was made using the independent-sample t-test. The average levels of lung function are displayed as mean±standard deviation, and the error bar in this figure represents the standard deviation. ^***^p<0.001.

Both FVC and FEV_1_ and their predicted values were higher in men than those in women (Table 2). In addition, participants’ FVC and FEV_1_ were higher than their predicted values. Similarly, most lung function indices, such as FVC, FEV_1_, VC, IC, RV, TV, ERV, IRV, TLC, PIF, and PEF, were significantly higher in men than those of women (Table 2). However, the ratio of FVC to FEV_1_ (FEV_1_/FVC) was higher in women than that in men (Table 2). Moreover, the FVC and FEV_1_ of participants were significantly lower in the obesity groups than in the non-obesity groups for all definitions of obesity (Figure 2) (Supplementary Material 4).

### Correlation between obesity parameters and lung function indicators

**Figure 3.**
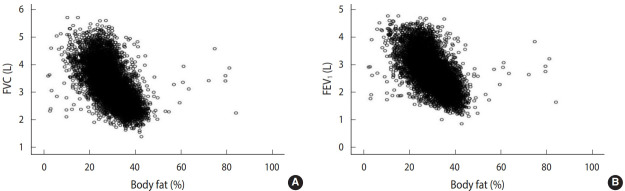
Spearman correlation analyses of the relationships among body fat percentage and lung function parameters such as FVC (A; Spearman ρ=-0.694, p<0.001) and FEV_1_ (B; Spearman ρ=-0.664, p<0.001) in the rural general adult Chinese population (n=8,284). FVC, forced vital capacity; FEV_1_, forced expiratory volume in 1 second.

Spearman correlation analyses demonstrated that several obesity parameters were significantly correlated with lung function levels (Table 3). BMI, WHtR, BFP, and VFI were negatively correlated with FVC and FEV_1_. However, WC, HC, WHR, and BM were positively correlated with FVC and FEV_1_ (Figure 3) (Supplementary Material 5). Both men and women displayed a similar direction of correlation between obesity parameters and lung function levels. In addition, BMI, WC, WHR, WHtR, BFP, BM, and VFI were negatively correlated with the ratio of FVC to FEV_1_ (FEV_1_/FVC), while height and HC were positively correlated with FEV_1_/FVC (Table 3). In addition, obesity parameters including BMI, WC, HC, WHR, WHtR, BFP, BM, and VFI were significantly correlated with most of the other lung function indicators, such as VC, IC, RV, TV, ERV, IRV, TLC, PIF, PEF, and PEFT (Supplementary Material 5). Remarkably, general obesity was more closely related to lung function in women than in men, while central obesity was more evident in men than in women (Table 3) (Supplementary Materials 5 and 6). Specifically, most obesity parameters such as BMI, WC, WHR, WHtR, and BFP were negatively correlated with lung function in women. However, this trend was not consistently evident in men. For example, in men, BMI was negatively correlated with FVC and FEV_1_, while WC was positively correlated with FVC and FEV_1_. However, both correlations were not significant in men, which is the most substantial difference when compared with women.

### Associations between obesity and lung function

The associations between obesity and lung function in men and women are shown in Table 4. After adjustment for their respective confounding factors in the full multiple linear regression model, we found that BMI, WHtR, and BFP were negatively associated with FVC and FEV_1_. In contrast, WC and WHR were positively associated with FVC and FEV_1_ (Table 4). Specifically, BMI, WHtR, and BFP were negatively associated with FVC (β_BMI_= -0.001, p < 0.001; β_WHtR_ = -0.525, p < 0.001; β_BFP_= -0.004, p < 0.001) and FEV_1_ (β_BMI_=-0.002, p<0.001; β_WHtR_ =-0.570, p<0.001; β_BFP_=-0.005, p< 0.001) in women. Similarly, WHtR and BFP were negatively associated with FVC (β_WHtR_ = -0.893, p< 0.001; β_BFP_= -0.003, p< 0.001) and FEV_1_ (β_WHtR_ = -0.754, p< 0.001; β_BFP_= -0.002, p< 0.001) in men. However, WC was positively associated with FVC (β_WC_ = 0.003, p< 0.001) and FEV_1_ (β_WC_ = 0.002, p< 0.001) in women. Additionally, WC and WHR were positively associated with FVC (β_WC_ = 0.003, p< 0.001; βWHR = 0.290, p< 0.001) and FEV_1_ (β_WC_ = 0.003, p< 0.001; βWHR = 0.162, p< 0.001) in men. The correlation and regression analyses showed consistent results regardless of gender (Tables 3 and 4).

Similarly, obesity defined by BMI, WHtR, and BFP was negatively associated with FVC and FEV_1_, whereas obesity defined by WC and WHR was positively associated with FVC and FEV_1_ (Table 4). Obesity defined by BMI, WHtR, and BFP was negatively associated with FVC (β_obesity defined by BMI_= -0.136, p< 0.001; β_obesity defined by WHtR_ = -0.049, p < 0.001; β_obesity defined by BFP_= -0.047, p < 0.001) and FEV_1_ (β_obesity defined by BMI_= -0.162, p< 0.001; β_obesity defined by WHtR_ = -0.048, p< 0.001; β_obesity defined by BFP_= -0.038, p< 0.001) in women. Likewise, obesity defined by BMI and WHtR was negatively associated with FVC (β_obesity defined by BMI_= -0.028, p< 0.001; β_obesity defined by WHtR_ =-0.069, p<0.001) and FEV_1_ (β_obesity defined by BMI_=-0.011, p=0.235; β_obesity defined by WHtR_ = -0.054, p< 0.001) in men. Nevertheless, WC and WHR were positively associated with FVC (β_obesity defined by WC_ = 0.057, p< 0.001; βWHR = 0.032, p< 0.001) and FEV_1_ (β_obesity defined by WC_=0.055, p<0.001; βWHR =0.005, p=0.089) in women. Meanwhile, WC and WHR were positively associated with FVC (β_obesity defined by WC_ =0.062, p< 0.001; βobesity defined by WHR = 0.027, p< 0.001) and FEV_1_ (β_obesity defined by WC_ =0.060, p<0.001; βobesity defined by WHR =0.017, p=0.092) in men.

## DISCUSSION

This study investigated the associations between obesity parameters and lung function in 8,284 adults from typical rural areas in central China. Our results showed a high prevalence of obesity, exceeding previously reported obesity rates in China. Although the prevalence of general obesity defined by height-related BMI and WHtR was not higher in women than in men, the prevalence of abdominal obesity defined by WC-related WC, WHR, and BFP was higher in women than in men. As expected, women had a lower level of lung function than men. Lung function was lower in the obesity groups than in the non-obesity groups for all definitions of obesity. Spearman correlation analyses showed that most of the obesity parameters were significantly correlated with the measured lung function indicators. After adjustment for potential confounders, WC-related parameters (e.g., WC and WHR) and obesity defined accordingly were positively associated with lung function parameters such as FVC and FEV_1_, while heightrelated obesity parameters (e.g., BMI, BFP, and WHtR) and obesity defined accordingly were negatively associated with lung function. Stratified by gender, overall, the association between general obesity and lung function was more evident in women than in men, while the link between central obesity and lung function was more obvious in men than in women.

Some previous studies have shown that obesity can alter chest wall mechanics, reduce lung volume, and increase airway resistance [4,11,13]. Nonetheless, inconclusive or inconsistent results have been reported regarding the associations between obesity parameters such as BMI and BFP and lung function indicators including FVC and FEV_1_, which may be due to previous studies’ small sample sizes, heterogeneous obesity parameters, and specific groups [19,21,44,45]. In this study, we found that several parameters (e.g., BMI, WC, WHR, WHtR, BFP, and VFI) representing incremental degrees of obesity were negatively associated with lung function indicators, which is consistent with the results of most previous studies. According to previous reports, height, HC, and BM are positively associated with lung function levels. Our results suggested that the obesity groups had lower lung function levels than the non-obesity groups. In other words, individuals with obesity tended to have smaller lung capacity and lower lung function.

Some recent studies have proposed that WC-related parameters such as WHR and WHtR might be better indicators than BMI to assess the role of obesity in predicting lung function in a general Caucasian population [44-46]. In this study, we investigated the association between several obesity-related parameters and a series of lung function indicators in the general Han Chinese population with a large sample. Spearman correlation analyses and linear regression analyses both showed that elevated BMI, WHR, WHtR, BFP, and obesity defined by BMI, WHR, WHtR, and BFP were associated with lower lung function levels, such as FVC and FEV_1_. However, increased height, BM, WC, HC, and obesity defined by WC were associated with higher FVC and FEV_1_.

There seem to be some inconsistencies in the relationships between several obesity-related parameters, as presented above, and lung function in this study. A careful comparison of them provides a reasonable explanation for the differences; that is, height, weight, WC, and HC are important factors related to lung function. Among them, height and WC are the 2 most important parameters in relation to lung function levels. Specifically, height is negatively related to certain obesity-related parameters such as BMI and WHtR, and obesity defined by BMI and WHtR was negatively associated with lung function because height is in the denominator of the formulas for BMI and WHtR and there is a positive relationship between height and lung function. Similarly, WHR is negatively related to HC, and obesity defined by WHR was inversely linked with lung function partly because HC is in the denominator in the formula for WHR and there is a positive relationship between HC and lung function. Notably, WC, WHR (as a parameter positively related to WC), and obesity defined by WC and WHR were positively associated with lung function, since WC is in the numerator in the formula of WHR, and there was a positive relationship between WC and lung function.

According to the results of Spearman correlation analyses and linear regression analyses, the order of the closeness of the correlations of obesity parameters with lung function was as follows: (1) height> weight> HC> WC; (2) WHtR> BFP> BMI> WHR. It is well known that the height of adults remains constant over time. Therefore, weight, HC, and WC are the obvious and crucial parameters related to obesity in individuals during adulthood. Previous studies have shown that BMI and BFP (for general obesity) and WHR and WHtR (for central obesity) are recommended to assess the relationship between obesity and lung function. WC and obesity defined by WC and WHR were positively associated with FVC and FEV_1_, which may indicate that lung function improved with the increase of WC to some degree, which is consistent with the results of previous studies [21,47,48]. However, height, weight, and HC have a stronger influence on lung function than WC, as manifested by the close relationships between obesity parameters, including WHtR, WHR, BMI, and lung function indicators such as FVC and FEV_1_.

Our findings indicate that several obesity-related parameters and obesity defined using those parameters were significantly associated with measures of lung function such as FVC and FEV_1_. The direction (inverse) of the effects of various obesity parameters (e.g., WHtR, WHR, BFP, and BMI) on lung function was similar, which suggests that obesity is significantly associated with lung damage. Systemic/limb/peripheral obesity defined by BMI, WHtR, and BFP was negatively associated with FVC and FEV_1_, while abdominal/belly/central obesity defined by WC and WHR was positively associated with FVC and FEV_1_. These results may suggest that taking only WC into account is inappropriate when investigating the relationship between obesity and lung function. In other words, it is better to define obesity using BMI, WHtR, and BFP than to use WC and WHR to evaluate their relationship with lung function.

It is worth mentioning that BMI was significantly associated with FEV_1_ in women, but not in men, according to linear regression analyses (Table 4), which is consistent with the higher correlation coefficient for BMI and lung function in women than in men (Table 3). In addition, this finding may be partly attributed to the larger sample size of women than that of men, as both men and women share the same direction of relationship between BMI and lung function. The finding of a positive association between WHR and lung function in men, but not in women, may be attributed to the higher WHR in men than in women (Table 2). A large WC and WC-related WHR may indicate a larger thoracic cavity and higher lung compliance and respiratory function [49]. Notably, there are some doubts regarding the stronger association of BFP and lung function in women than in men (Table 4). This is consistent with the higher BFP and correlation coefficients of BFP, WC, WHR, WHtR, VFI, and lung function in women than in men. Additionally, BFP is closely linked to subcutaneous and visceral fat, which mainly accumulates in the waist and abdomen. Compared with men, most rural women have a lower education and family income level, as well as birthing experience and subsequent body alterations, which may lead to a higher BFP than in men, in combination with a poor awareness of weight control and loss. There are differences in the distribution and level of BFP and its related parameters of muscle mass and strength between men and women; these differences may influence the contractility of respiratory muscles and subsequent respiratory function [50].

There were several strengths of our study. First, we recruited a large population sample of adults across a broad age range from a typical rural area in central China, which enabled us to derive insights into the prevalence of obesity and lung function levels in the rural area. Second, the analysis of several obesity parameters made it possible to comprehensively investigate their associations with lung function to obtain more accurate and detailed information. Third, the large sample size, assessment of potential confounders, and several effect models facilitated the investigation of the independent effects of peripheral and central obesity on lung function. However, several limitations of this study should be acknowledged. First, the cross-sectional nature of this study makes it difficult to draw causal conclusions between obesity and lung function. Second, although several important factors were taken into account in the analyses, there remains a possibility of residual confounding caused by other unmeasured variables, such as muscle mass and strength, metabolic factors, markers of inflammation, nutritional status, and history or indicators of allergy. Third, it is necessary to determine whether these results are generalizable to other ethnic populations for further investigation.

In summary, both general and central obesity are negatively associated with lung function in the general Chinese rural adult population. There was an inverse effect of WC-related WHR and height-related WHtR on lung function. In particular, height-related obesity parameters, such as BMI and WHtR, had negative associations with lung function, whereas WC-related obesity parameters, such as WC and WHR, had positive associations with lung function. Given the inverse association between BFP and lung function, BMI (for general obesity) and WHtR (for central obesity) may be the preferred parameters to assess the relationship between obesity and lung function in the general population. Individuals with obesity and higher values of obesity-related parameters had lower lung function levels. Compared with men, the exacerbation of obesity was more closely associated with declining lung function in women. However, the underlying mechanism between these relationships needs further investigation.

## Figures and Tables

**Table 1.** Demographic characteristics of the study population

Characteristics	Men (n=3,327)	Women (n=4,957)	p-value
Age, mean±SD (yr)	52.12±12.42	51.23±12.09	0.009^1^
Smoking			<0.001^2^
Never smokers	1,095 (32.9)	4,904 (98.9)	
Ex-smokers/former smokers	586 (17.6)	13 (0.3)	
Current smokers	1,645 (49.5)	39 (0.8)	
Drinking			<0.001^2^
Never drinkers	1,336 (40.2)	4,848 (97.8)	
Ex-drinkers/former drinkers	294 (8.8)	11 (0.2)	
Current drinkers	1,696 (51.0)	97 (2.0)	
Family monthly income level per capita (Chinese yuan)			<0.001^2^
<500	971 (29.2)	1,569 (31.7)	
500-999	1,183 (35.6)	1,804 (36.4)	
1,000-1,999	735 (22.1)	1,103 (22.3)	
2,000-2,999	238 (7.2)	294 (6.0)	
≥3,000	197 (5.9)	175 (3.5)	
Education level			<0.001^2^
Illiteracy	115 (3.5)	599 (12.1)	
Primary school	577 (17.3)	1,073 (21.6)	
Middle school	1,490 (44.8)	1,988 (40.1)	
Secondary school or high school	890 (26.8)	953 (19.2)	
College/university and above	254 (7.6)	341 (6.9)	

Values are presented as number (%). SD, standard deviation.

1 Analysis by the independent-sample t-test.

2 Analysis by the chi-square test.

**Table 2.** Levels of obesity and lung function parameters in the adult population from Qiliying and Langgongmiao in Xinxiang County, China

Characteristics	Men (n=3,327)	Women (n=4,957)	p-value
Lung function parameters			
FVC (L)	3.87±0.50	2.72±0.40	<0.001^1^
FVC predicted (L)	3.73±0.49	2.67±0.34	<0.001^1^
FEV_1_ (L)	3.18±0.54	2.34±0.43	<0.001^1^
FEV_1_ predicted (L)	2.98±0.47	2.16±0.33	<0.001^1^
FEV_1_/FVC	0.81 (0.79-0.84)	0.85 (0.83-0.87)	<0.001^2^
VC (L)	3.68±0.34	2.64±0.27	<0.001^1^
IC (L)	2.12±0.73	1.64±0.61	<0.001^1^
RV (L)	1.56±0.15	1.59±0.18	<0.001^1^
TV (L)	0.61 (0.43-0.84)	0.48 (0.33-0.67)	<0.001^2^
ERV (L)	0.93 (0.58-1.30)	0.56 (0.32-0.82)	<0.001^2^
IRV (L)	1.45 (1.07-1.87)	1.15 (0.80-1.45)	<0.001^2^
TLC (L)	5.57±0.34	4.04±0.28	<0.001^1^
RV/TLV (%)	30.4 (26.9-33.7)	28.9 (26.5-31.0)	<0.001^2^
PIF (L/sec)	2.08 (1.55 -2.76)	1.79 (1.36-2.33)	<0.001^2^
PEF (L/sec)	8.40 (7.94-8.87)	5.96 (5.65-6.29)	<0.001^2^
PEFT (sec)	0.25 (0.16-0.42)	0.27 (0.18-0.42)	<0.001^2^
Physical parameters			
Height (cm)	168.66±6.43	157.44±5.88	<0.001^1^
Weight (kg)	72.90±11.51	63.29±10.02	<0.001^1^
BMI (kg/m^2^)	25.60 (23.14-28.03)	25.16 (22.96-27.44)	0.106^2^
WC (cm)	91.39±7.02	87.19±9.36	<0.001^1^
HC (cm)	98.03±6.56	97.23±7.49	<0.001^1^
WHR	0.914 (0.867-0.960)	0.867 (0.813-0.922)	<0.001^2^
WHtR	0.533 (0.491-0.572)	0.539 (0.490-0.585)	0.002^2^
BFP	25.6 (22.2-28.8)	34.1 (30.6-37.0)	<0.001^2^
BM (kcal)	1,626.01±188.68	1,304.15±165.55	<0.001^1^
VFI	12.43±5.11	8.47±4.51	<0.001^1^

Values are presented as mean±standard deviation (normal distribution data) or median values with interquartile (non-normal distribution data). FVC, forced vital capacity; FEV1, forced expiratory volume in 1 second; VC, vital capacity; IC, inspiratory capacity; RV, residual volume; TV, tidal volume; ERV, expiratory reserve volume; IRV, inspiratory reserve volume; TLC, total lung capacity; TLV, total lung volume; PIF, peak inspiratory flow; PEF, peak expiratory flow; PEFT, peak expiratory flow time; BMI, body mass index; WC, waist circumference; HC, hip circumference; WHR, waist-to-hip ratio; WHtR, waist-to-height ratio; BFP, body fat percentage; BM, basal metabolism; VFI, visceral fat index.

1 Analysis by the independent-sample t-test.

2 Analysis by the Mann-Whitney U test.

**Table 3.** Spearman correlation coefficients between obesity parameters and lung function indicators in the adult population from Xinxiang County, China (n=8,284)

Variables	Men			Women		
	FVC	FEV_1_	FEV_1_/FVC	FVC	FEV_1_	FEV_1_/FVC
Height	0.742^***^	0.713^***^	0.357^***^	0.718^***^	0.709^***^	0.503^***^
Weight	0.329^***^	0.333^***^	0.210^***^	0.166^***^	0.143^***^	0.063^***^
BMI	-0.018	-0.001	-0.051^***^	-0.172^***^	-0.193^***^	-0.178^***^
WC	0.033	0.033	0.016	-0.234^***^	-0.265^***^	-0.285^***^
HC	0.148^***^	0.185^**^	0.177^***^	0.083^***^	0.084^***^	0.074^***^
WHR	-0.077^***^	-0.107^***^	-0.122^***^	-0.377^***^	-0.419^***^	-0.436^***^
WHtR	-0.208^***^	-0.199^***^	-0.097^***^	-0.429^***^	-0.456^***^	-0.412^***^
BFP	-0.229^***^	-0.249^***^	-0.216^***^	-0.470^***^	-0.500^***^	-0.469^***^
BM	0.410^***^	0.422^***^	0.291^***^	0.270^***^	0.250^***^	0.165^***^
VFI	-0.233^***^	-0.240^***^	-0.184^***^	-0.370^***^	-0.400^***^	-0.377^***^

FVC, forced vital capacity; FEV1, forced expiratory volume in 1 second; BMI, body mass index; WC, waist circumference; HC, hip circumference; WHR, waist-to-hip ratio; WHtR, waist-to-height ratio; BFP, body fat percentage; BM, basal metabolism; VFI, visceral fat index.

** p≤0.01,

*** p≤0.001.

**Table 4.** Associations between obesity and lung function (n=8284)

Obesity parameters		Men				Women			
		β	FVC (L)	β	FEV_1_ (L)	β	FVC (L)	β	FEV_1_ (L)
			B (95% CI for B)		B (95% CI for B)		B (95% CI for B)		B (95% CI for B)
BMI									
	Univariate model	-0.016	-0.002 (-0.007, 0.003)	-0.005	-0.001(-0.006, 0.005)	-0.156	-0.016 (-0.019, -0.013)^***^	-0.187	-0.021 (-0.025, -0.018)^***^
	Full model	-0.023	-0.003 (-0.007, 0.000)^#^	-0.013	-0.002 (-0.005, 0.001)	-0.009	-0.001 (-0.003, 0.001)	-0.019	-0.002 (-0.004, -0.001)^***^
WC									
	Univariate model	0.038	0.002 (0.000, 0.004)^***^	0.026	0.001 (0.000, 0.003)^***^	-0.203	-0.008 (-0.009, -0.007)^***^	-0.251	-0.010 (-0.011, -0.009)^***^
	Full model	0.069	0.003 (0.002, 0.005)^***^	0.065	0.003 (0.003, 0.004)^***^	0.072	0.003 (0.002, 0.004)^***^	0.062	0.002 (0.002, 0.003)^***^
WHR									
	Univariate model	-0.077	-0.549 (-0.791, -0.306)^***^	-0.125	-0.964 (-1.224, -0.703)^***^	-0.299	-1.272 (-1.386, -1.159)^***^	-0.345	-1.645 (-1.767, -1.524)^***^
	Full model	0.040	0.290 (0.110, 0.470)^***^	0.021	0.162 (0.024, 0.300)^***^	0.012	0.051 (-0.036, 0.138)	-0.002	-0.007 (-0.083, 0.069)
WHtR									
	Univariate model	-0.214	-1.772 (-2.047, -1.497)^***^	-0.218	-1.949 (-2.245, -1.653)^***^	-0.402	-2.248 (-2.391, -2.105)^***^	-0.448	-2.730 (-2.882, -2.578)^***^
	Full model	-0.108	-0.893 (-1.098, -0.688)^***^	-0.084	-0.754 (-0.910, -0.597)^***^	-0.094	-0.525 (-0.640, -0.410)^***^	-0.093	-0.570 (-0.671, -0.470)^***^
BFP									
	Univariate model	-0.220	-0.020 (-0.023, -0.017)^***^	-0.255	-0.025 (-0.028, -0.022)^***^	-0.419	-0.032 (-0.034, -0.030)^***^	-0.472	-0.039 (-0.041, -0.037)^***^
	Full model	-0.029	-0.003 (-0.005, 0.000)^***^	-0.016	-0.002 (-0.003, 0.000)^***^	-0.049	-0.004 (-0.005, -0.002)^***^	-0.055	-0.005 (-0.006, -0.003)^***^
Obesity defined by BMI									
	Univariate model	-0.023	-0.024 (-0.058, 0.011)	-0.005	-0.006 (-0.043, 0.031)	-0.136	-0.109 (-0.131, -0.087)^***^	-0.162	-0.141 (-0.165, -0.117)^***^
	Full model	-0.028	-0.028 (-0.053, -0.003)^***^	-0.011	-0.012 (-0.031, 0.008)	-0.005	-0.004 (-0.019, 0.011)	-0.011	-0.010 (-0.023, 0.003)
Obesity defined by WC									
	Univariate model	0.040	0.041 (0.006, 0.075)^***^	0.032	0.035 (-0.002, 0.072)^#^	-0.170	-0.142 (-0.165, -0.119)^***^	-0.215	-0.196 (-0.221, -0.172)^***^
	Full model	0.062	0.062 (0.038, 0.087)^***^	0.060	0.065 (0.046, 0.084)^***^	0.068	0.057 (0.041, 0.073)^***^	0.055	0.050 (0.036, 0.064)^***^
Obesity defined by WHR									
	Univariate model	-0.060	-0.061 (-0.096, -0.027)^***^	-0.093	-0.103 (-0.140, -0.065)^***^	-0.241	-0.241 (-0.268, -0.214)^***^	-0.303	-0.331 (-0.360, -0.302)^***^
	Full model	0.027	0.027 (0.002, 0.053)^***^	0.015	0.017 (-0.003, 0.036)	0.032	0.032 (0.013, 0.052)^***^	0.004	0.005 (-0.013, 0.022)
Obesity defined by WHtR									
	Univariate model	-0.153	-0.167 (-0.204, -0.130)^***^	-0.159	-0.189 (-0.227, -0.147)^***^	-0.319	-0.275 (-0.298,-0.252)^***^	-0.360	-0.338 (-0.363, -0.314)^***^
	Full model	-0.069	-0.076 (-0.103, -0.049)^***^	-0.054	-0.064 (-0.085, -0.043)^***^	-0.049	-0.042 (-0.059, -0.025)^***^	-0.051	-0.048 (-0.063, -0.033)^***^
Obesity defined by BFP									
	Univariate model	-0.180	-0.182 (-0.216, -0.148)^***^	-0.202	-0.220 (-0.256, -0.183)^***^	-0.373	-0.302 (-0.323, -0.281)^***^	-0.413	-0.365 (-0.387, -0.342)^***^
	Full model	-0.018	-0.019 (-0.044, 0.007)	0.001	0.001 (-0.020, 0.019)	-0.047	-0.038 (-0.055, -0.022)^***^	-0.043	-0.038 (-0.052, -0.023)^***^

B, unstandardized beta; β, standardized beta; FVC, forced vital capacity; FEV1, forced expiratory volume in 1 second; BMI, body mass index; WC, waist circumference; WHR, waist-to-hip ratio; WHtR, waist-to-height ratio; BFP, body fat percentage.

1 The obesity parameters were independent variables, while lung function indicators were dependent variables; Full model: adjusted for age, gender, smoking, drinking, family income, and education level.

# 0.05<p≤0.10,

*** p ≤0.001.

## SUPPLEMENTARY MATERIALS

Supplementary Material 1. The location of the sampling sites in Xinxiang county (n = 8,284). Both Qiliying and Langgongmiao towns are located on Xinxiang county, and the distance between the two towns is approximate 10 kilometers.

Supplementary Material 2. The distribution of BMI (A) and BFP (B) among the subjects (n = 8,284). BMI: Body mass index. BFP: Body Fat Percentage. ^***^: p < 0.001.

Supplementary Material 3. Comparison in percentage of obesity between men (n = 3,327) and women (n = 4,957).

Supplementary Material 4. Comparison of lung function between obesity and non-obesity groups defined by different obesity parameters in men and women

Supplementary Material 5. Spearman correlation coefficients between obesity parameters and lung function indicators in adult population from Xinxiang (n = 8284).

Supplementary Material 6. Spearman correlation analyses of the relationship among body fat percentage and lung function parameters such as FVC (Figure 3A, men, spearman’s ρ = -0.229, p < 0.001; Figure S3B, women, spearman’s ρ = -0.470, p < 0.001) and FEV1 (Figure S3C, men, spearman’s ρ = -0.249, p < 0.001; Figure S3D, women, spearman’s ρ = -0.500, p < 0.001) in men (n = 3,327) and women (n = 4,957) from the Chinese rural areas.
