# Supplementary material for: Obesity parameters in relation to lung function levels in a large Chinese rural adult population
Source: Epidemiol Health. 2021 Aug 3;43:e2021047. doi: 10.4178/epih.e2021047 (PMC8602009; doi:10.4178/epih.e2021047)
Supplement: Supplementary Material 1. — The location of the sampling sites in Xinxiang county (n = 8,284). Both Qiliying and Langgongmiao towns are located on Xinxiang county, and the distance between the two towns is approximate 10 kilometers. [file epih-43-e2021047-suppl1.pdf]

Volume: 43, Article ID: e2021047
https://doi.org/10.4178/epih.e2021047

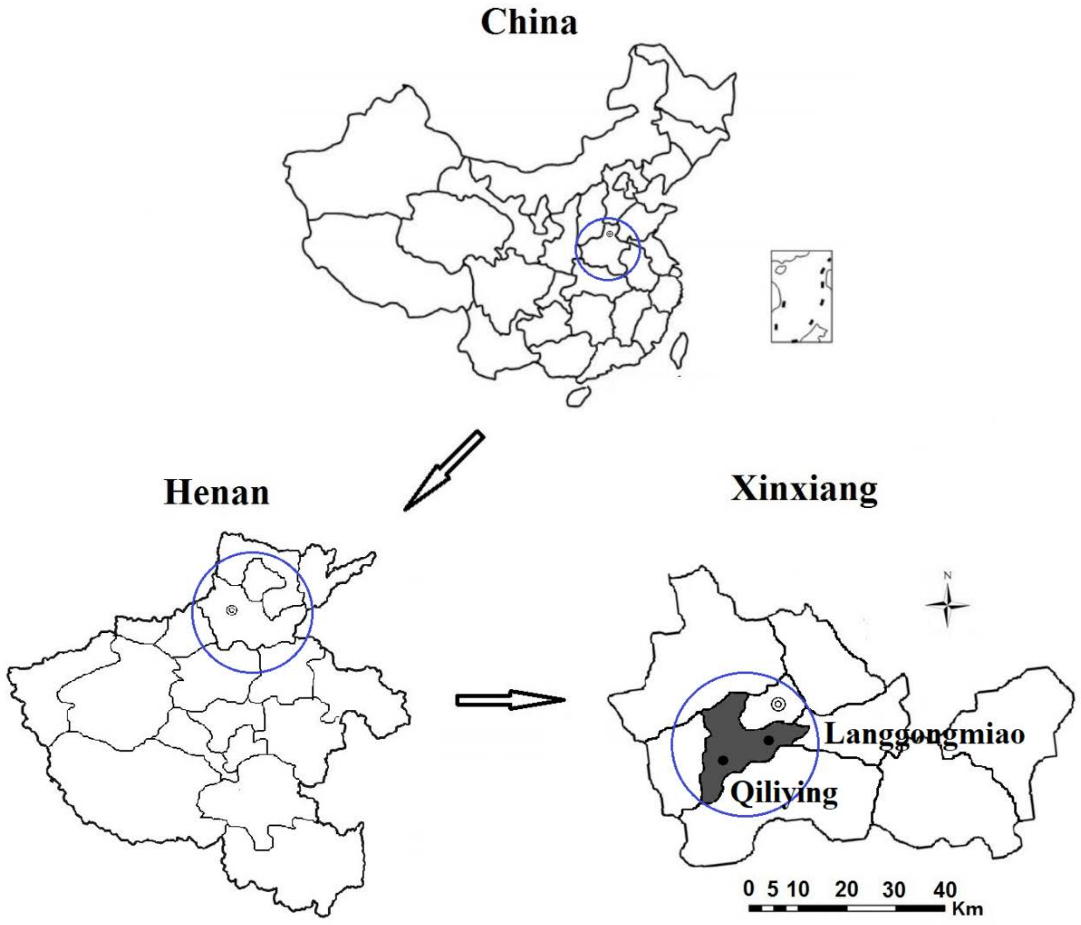

Supplementary Material 1. The location of the sampling sites in Xinxiang county (n = 8,284). Both Qiliying and Langgongmiao towns are located on Xinxiang county, and the distance between the two towns is approximate 10 kilometers.
