# Supplementary material for: Obesity parameters in relation to lung function levels in a large Chinese rural adult population
Source: Epidemiol Health. 2021 Aug 3;43:e2021047. doi: 10.4178/epih.e2021047 (PMC8602009; doi:10.4178/epih.e2021047)
Supplement: Supplementary Material 2. — The distribution of BMI (A) and BFP (B) among the subjects (n = 8,284). BMI: Body mass index. BFP: Body Fat Percentage. ***: p < 0.001. [file epih-43-e2021047-suppl2.pdf]

Volume: 43, Article ID: e2021047
https://doi.org/10.4178/epih.e2021047

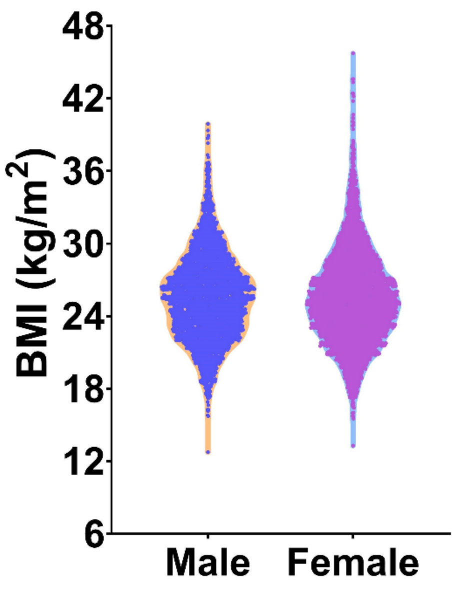

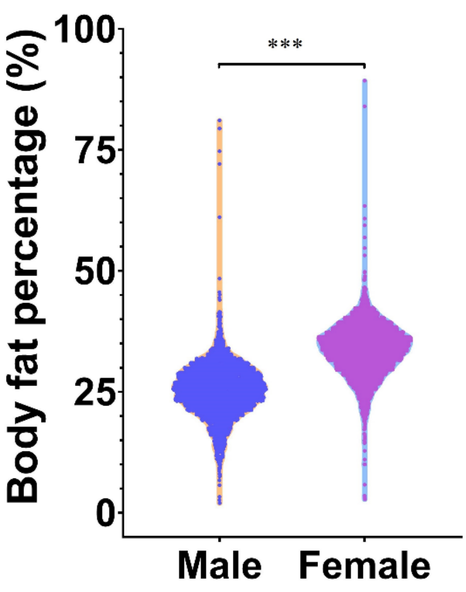

Supplementary Material 2. The distribution of BMI (A) and BFP (B) among the subjects (n = 8,284). BMI: Body mass index. BFP: Body Fat Percentage. ***: $p < 0.001$.
