# Supplementary material for: Obesity parameters in relation to lung function levels in a large Chinese rural adult population
Source: Epidemiol Health. 2021 Aug 3;43:e2021047. doi: 10.4178/epih.e2021047 (PMC8602009; doi:10.4178/epih.e2021047)
Supplement: Supplementary Material 3. — Comparison in percentage of obesity between men (n = 3,327) and women (n = 4,957). [file epih-43-e2021047-suppl3.pdf]

https://doi.org/10.4178/epih.e2021047

Supplementary Material 3. Comparison in percentage of obesity between men (n = 3,327) and women (n = 4,957).

Obesity (n, %)	Men (n = 3,327)	Women (n = 4,957)	p-value
Defined on BMI	1,858 (55.8)	2,631 (53.1)	0.013[c]
Defined on WC	1,597 (48.0)	3,222 (65.0)	< 0.001[c]
Defined on WHR	1,944 (58.4)	3,975 (80.2)	< 0.001[c]
Defined on WHtR	2,311 (69.5)	3,421 (69.1)	0.679[c]
Defined on BFP	1,788 (53.7)	2,890 (58.3)	< 0.001[c]

Abbreviation, BMI: Body Mass Index; WC: waist circumference; WHR: Waist circumference to hip circumference ratio; WHtR: waist circumference to height ration; BFP: Body fat percentage.
