# Supplementary material for: Obesity parameters in relation to lung function levels in a large Chinese rural adult population
Source: Epidemiol Health. 2021 Aug 3;43:e2021047. doi: 10.4178/epih.e2021047 (PMC8602009; doi:10.4178/epih.e2021047)
Supplement: Supplementary Material 4. — Comparison of lung function between obesity and non-obesity groups defined by different obesity parameters in men and women [file epih-43-e2021047-suppl4.pdf]

Volume: 43, Article ID: e2021047
https://doi.org/10.4178/epih.e2021047

Supplementary Material 4. Comparison of lung function between obesity and non-obesity groups defined by different obesity parameters in men and women.

	FVC (L)			FEV_1 (L)		
	Obesity	Non obesity	p-value	Obesity	Non obesity	p-value
Obesity groups defined by different parameters in men						
BMI	3.89 ± 0.53	3.86 ± 0.48	0.200	3.19 ± 0.57	3.19 ± 0.52	0.251
WC	3.85 ± 0.52	3.89 ± 0.48	0.404	3.17 ± 0.57	3.21 ± 0.51	0.152
WHR	3.85 ± 0.49	3.91 ± 0.52	0.106	3.15 ± 0.52	3.25 ± 0.57	0.006
WHtR	3.82 ± 0.48	3.99 ± 0.53	0.003	3.13 ± 0.52	3.32 ± 0.57	< 0.001
BFP	3.79 ± 0.48	3.97 ± 0.51	0.015	3.09 ± 0.52	3.31 ± 0.54	< 0.001
Obesity groups defined by different parameters in women						
BMI	2.67 ± 0.38	2.78 ± 0.41	< 0.001	2.27 ± 0.41	2.41 ± 0.45	< 0.001
WC	2.67 ± 0.39	2.82 ± 0.40	< 0.001	2.27 ± 0.42	2.47 ± 0.44	< 0.001
WHR	2.68 ± 0.39	2.92 ± 0.36	< 0.001	2.27 ± 0.42	2.60 ± 0.40	< 0.001
WHtR	2.64 ± 0.37	2.91 ± 0.39	< 0.001	2.23 ± 0.40	2.57 ± 0.41	< 0.001
BFP	2.60 ± 0.36	2.90 ± 0.39	< 0.001	2.19 ± 0.39	2.55 ± 0.40	< 0.001

Abbreviation, FVC: forced vital capacity; FEV_1: forced expiratory volume in 1 second; BMI: Body Mass Index; WHR: the ratio of waist circumference to hip circumference; WHtR: the ratio of waist circumference to height; BFP: Body fat percentage; Obesity was defined BMI

Volume: 43, Article ID: e2021047
https://doi.org/10.4178/epih.e2021047

using as a BMI ⩾ 25 kg/m^2; Obesity was defined by WC using as WC ⩾ 90 cm for men and WC ⩾ 80 cm for women; Obesity was defined by WHR using as WHR ⩾ 0.90 for men and WHR ⩾ 0.80 for women; Obesity was defined by WHtR using as WHtR ⩾ 0.5 both for men and women; Obesity is defined BFP using as BFP ⩾ 25 for men and BFP ⩾ 33 for women. Comparison of FVC and FEV_1 between obesity and non-obesity were used by independent-sample t-test.
