# Supplementary material for: Obesity parameters in relation to lung function levels in a large Chinese rural adult population
Source: Epidemiol Health. 2021 Aug 3;43:e2021047. doi: 10.4178/epih.e2021047 (PMC8602009; doi:10.4178/epih.e2021047)
Supplement: Supplementary Material 5. — Spearman correlation coefficients between obesity parameters and lung function indicators in adult population from Xinxiang (n = 8284). [file epih-43-e2021047-suppl5.pdf]

Volume: 43, Article ID: e2021047
https://doi.org/10.4178/epih.e2021047

Supplementary Material 5. Spearman correlation coefficients between obesity parameters and lung function indicators in adult population from Xinxiang (n = 8284).

	FVC	FEV$_1$	VC	IC	RV	TV	ERV	IRV	TLC	RV/TLC	PIF	PEF
**Men**												
Height	0.742***	0.713***	0.695***	0.342***	0.429***	0.126***	0.265***	0.316***	0.892***	-0.438***	0.153***	0.757***
Weight	0.329***	0.333***	0.347***	0.346***	0.193***	0.139***	-0.094***	0.299***	0.425***	-0.463***	0.186***	0.398***
BMI	-0.018	0.001	0.026	0.205***	-0.017	0.093***	-0.252***	0.166***	0.011	-0.286***	0.126***	0.048**
WC	0.033#	0.033#	0.056***	0.205***	0.160***	0.104***	-0.239***	0.162***	0.108***	-0.235***	0.133***	0.112***
HC	0.148***	0.185***	0.217***	0.230***	0.112***	0.110***	-0.114***	0.186***	0.263***	-0.360***	0.178***	0.280***
WHR	-0.077***	-0.107**	-0.099***	0.108***	0.136***	0.057***	-0.249***	0.082***	-0.064***	-0.048***	0.043*	-0.071***
WHtR	-0.208***	-0.199***	-0.170***	-0.090***	0.014	0.063	-0.329***	0.055***	-0.178***	0.096***	0.079***	-0.133***
BFP	-0.229***	-0.249***	-0.237***	0.038*	0.194***	0.033#	-0.326***	0.011	-0.180***	0.057***	0.033#	0.185***
BM	0.410***	0.422***	0.432***	0.370***	0.167***	0.141***	-0.031#	0.326***	0.504***	-0.527***	0.196***	0.475***
VFI	-0.223***	-0.240***	-0.217***	0.098***	0.141***	0.062***	-0.314***	0.063***	-0.187***	-0.016	0.067***	-0.174***
**Women**												
Height	0.718***	0.709***	0.662***	0.305***	0.786***	0.117***	0.235***	0.267***	0.857***	0.095***	0.161***	0.780***
Weight	0.166***	0.143***	0.122***	0.225***	0.379***	0.080***	-0.165***	0.180***	0.224***	0.200***	0.123***	0.195***
BMI	-0.172***	-0.193***	-0.192***	0.080***	0.008	0.023	-0.289***	0.050***	-0.177***	0.157***	0.047***	-0.172***
WC	-0.234***	-0.265***	-0.283***	0.035*	0.215***	0.018	-0.301***	-0.002	-0.192***	0.383***	0.042**	-0.215***
HC	0.083***	0.084***	0.079***	0.153***	0.231***	0.068***	-0.125***	0.118***	0.139***	0.114***	0.104***	0.105***
WHR	-0.377***	-0.419***	-0.438***	-0.065***	0.132***	-0.025#	-0.300***	-0.086***	-0.357***	0.438***	-0.013	-0.366***

https://doi.org/10.4178/epih.e2021047

WHtR	-0.429***	-0.456***	-0.459***	-0.058***	-0.011	-0.016	-0.357***	-0.082***	-0.423***	0.336***	-0.006	-0.427***
BFP	-0.470***	-0.500***	-0.507***	-0.078***	0.041**	-0.048***	-0.357***	-0.089***	-0.450***	0.416***	-0.016	-0.463***
BM	0.270***	0.250***	0.225***	0.254***	0.416***	0.094***	-0.108***	0.207***	0.328***	0.142***	0.131***	0.300***
VFI	-0.370***	-0.400***	-0.404***	-0.004	0.026$^{\#}$	-0.002	-0.340***	-0.029*	-0.362***	0.334***	0.010	-0.365***

The upper part of the table is based on men according to Spearman correlation analysis results; The lower part of the table is based on women according to Spearman correlation analysis results. Abbreviation, FVC: forced vital capacity; FEV_1: forced expiratory volume in 1 second; VC: Vital capacity; IC: Inspiratory capacity; RV: Residual volume; TV: Tidal volume; ERV: Expiratory reserve volume; IRV: Inspiratory reserve volume; TLC: Total lung capacity; PIF: Peak inspiratory flow; PEF: Peak expiratory flow; PEFT: Peak expiratory flow time; BMI: Body Mass Index; WHR: the ratio of waist circumference to hip circumference; WHtR: the ratio of waist circumference to height; BFP: Body fat percentage; BM: Basal metabolism; VFI: Visceral fat index. $^{\#}$: $0.05 < p < 0.10$; *: $p < 0.05$; **: $p < 0.01^{**}$; ***: $p < 0.001$.
