# Supplementary material for: Obesity parameters in relation to lung function levels in a large Chinese rural adult population
Source: Epidemiol Health. 2021 Aug 3;43:e2021047. doi: 10.4178/epih.e2021047 (PMC8602009; doi:10.4178/epih.e2021047)
Supplement: Supplementary Material 6. — Spearman correlation analyses of the relationship among body fat percentage and lung function parameters such as FVC (Figure 3A, men, spearman’s ρ = -0.229, p < 0.001; Figure S3B, women, spearman’s ρ = -0.470, p < 0.001) and FEV1 (Figure S3C, men, spearman’s ρ = -0.249, p < 0.001; Figure S3D, women, spearman’s ρ = -0.500, p < 0.001) in men (n = 3,327) and women (n = 4,957) from the Chinese rural areas. [file epih-43-e2021047-suppl6.pdf]

https://doi.org/10.4178/epih.e2021047

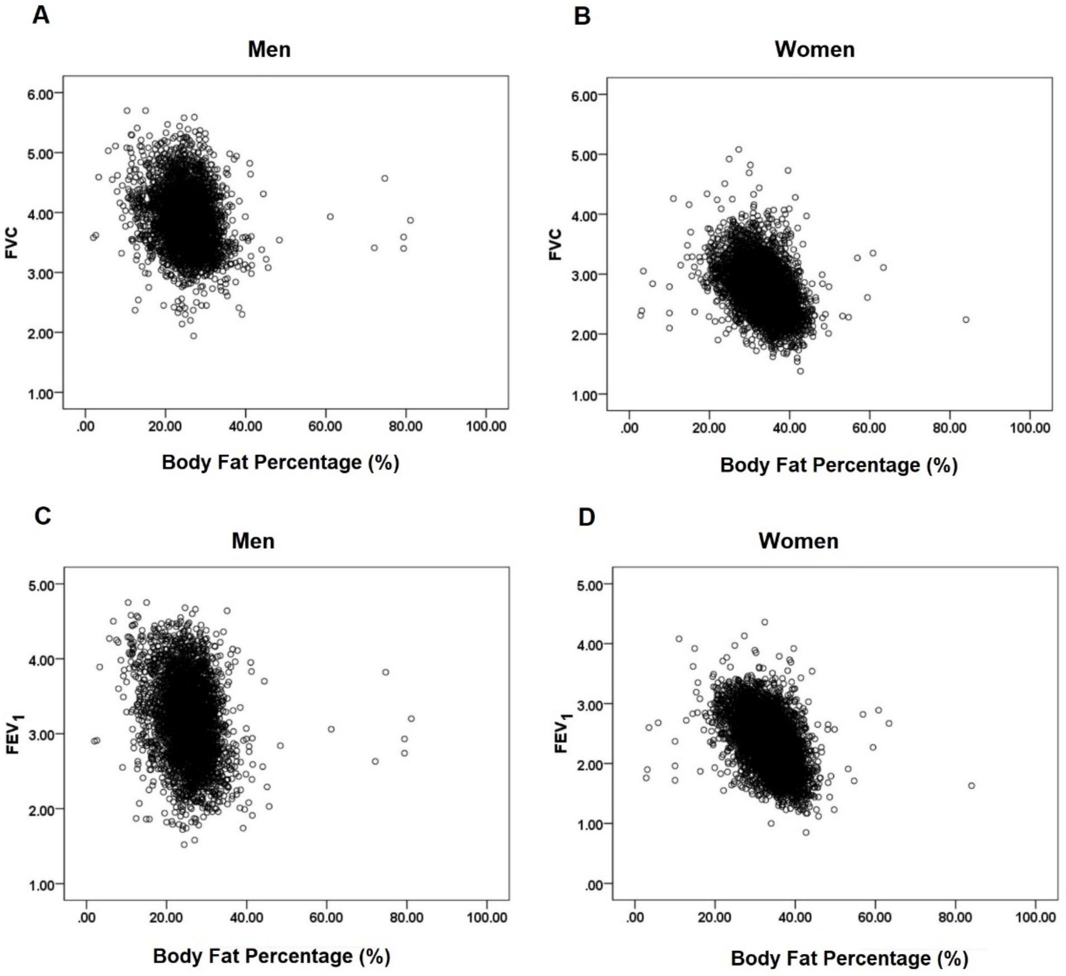


**Supplementary Material 6.** Spearman correlation analyses of the relationship among body fat percentage and lung function parameters such as FVC (Figure 3A, men, spearman's ρ = -0.229, p < 0.001; Figure S3B, women, spearman's ρ = -0.470, p < 0.001) and FEV1 (Figure S3C, men, spearman's ρ = -0.249, p < 0.001; Figure S3D, women, spearman's ρ = -0.500, p < 0.001) in men (n = 3,327) and women (n = 4,957) from the Chinese rural areas.
